# A Participatory-Based Research Approach for Assessing Exposure to Lead-Contaminated Drinking Water in the Houston Neighborhood of the Greater Fifth Ward

**DOI:** 10.3390/ijerph19138135

**Published:** 2022-07-02

**Authors:** Leanne S. Fawkes, Thomas J. McDonald, Taehyun Roh, Weihsueh A. Chiu, Robert J. Taylor, Garett T. Sansom

**Affiliations:** 1 Department of Environmental and Occupational Health, School of Public Health, Texas A&M University, College Station, TX 77843, USA; t-mcdonald12332@tamu.edu (T.J.M.); sansom@tamu.edu (G.T.S.); 2 Department of Epidemiology & Biostatistics, School of Public Health, Texas A&M University, College Station, TX 77843, USA; taehyunroh@tamu.edu; 3 Veterinary Integrative Biosciences, Veterinary Medicine & Biomedical Sciences, Texas A&M University, College Station, TX 77843, USA; wchiu@cvm.tamu.edu (W.A.C.); rtaylor@cvm.tamu.edu (R.J.T.)

**Keywords:** environmental health, drinking water, lead contamination, environmental justice, participatory-based research

## Abstract

To address community-driven concerns about lead-contaminated drinking water in residential homes in the Greater Fifth Ward neighborhood in Northeast Houston, Texas utilizing participatory-based research. The study collected survey data and performed lead analysis on drinking water from residents’ homes. The Greater Fifth Ward is characterized as a majority-minority environmental justice community and is located within two confirmed cancer clusters. The residents of 172 homes completed a survey and had detectable lead levels in their water samples. Survey results indicated that more than half of the residents (58.2%) were concerned with the water quality and 42.9% rated the drinking water as poor. Water lead levels detected ranged from 0.01 to 22 µg/L. 10.9% of homes exceeding 1 µg/L, and one located exceeded the USEPA’s action limit of 15 µg/L. Homes built prior to 1978 without major renovation had significantly higher levels of lead in their drinking water compared to homes built after 1978 (*p*-value < 0.05). These findings demonstrate the need for lead testing of residential water in low socioeconomic-status communities, as well as demonstrating the benefits of community engagement and participatory research to address environmental health concerns.

## 1. Introduction

Lead is a persistent pollutant in water and does not degrade. Lead exposure is associated with serious adverse health effects in humans, such as reduction in cognitive functioning, heart disease, organ damage, and nervous system damage [1]. While lead is a naturally occurring element, it can enter drinking water through both natural and anthropogenic sources [2]. Most lead exposures are related to anthropogenic sources, such as mining, smelting, and other industrial uses [3]. Concentrations of high lead levels in tap water are often attributable to the corrosion of solder and pipe connections within homes or within municipal water lines that supply the home [4]. Routes of exposure to the human body are inhalation (30% to 50% absorbed in the bloodstream), followed by ingestion (8% to 15% absorbed in the bloodstream), and dermal absorption [2]. Yet, the main route of exposure to children is generally through the ingestion of lead paint in homes built pre-1978 [5]. Associated adverse health outcomes from lead exposure are contingent on the levels of exposure and life stage. The population at greatest risk to lead exposure are children aged five years and younger [6]. Lower exposure levels can negatively impact a child’s cognitive and behavioral functioning because their nervous systems are still developing [7,8]. Other adverse health effects of childhood lead exposure include damage to the brain, delayed growth and development, learning, hearing, and speech problems [6]. Racial disparities of lead exposure disproportionately affecting black children have been well documented [9,10,11]. Studies that examined grade school intelligence quotient (IQ) with elevated blood lead levels (BLL) at ≥2 μg/dL revealed that black infants lost 1.78 IQ points, which is 0.27–0.63 more IQ points than white or Hispanic infants (1.78 IQ points vs. −1.15 and −1.21) [12]. Those with a BLL of ≥2 μg/dL were assessed because a BLL of ≥2 μg/dL during early childhood is shown to result in a loss of IQ that is noticeable in grade school [12,13,14]. The Centers for Disease Control and Prevention (CDC) blood lead reference value (BLRV) is 3.5 μg/dL, which helps identify those children with blood lead levels that are higher than most children’s levels and is based on NHANES data [15]. High-risk populations, such as low-income, urban neighborhoods, minorities, and those that live in homes built before 1978, have an increased risk of lead exposure [16,17].

Lead-containing components, such as lead service lines, leaded pipes, solders, and faucets, can release lead into the water [18]. Although lead pipes were not generally used after 1930, lead soldering was still common until 1986 [19]. While there is no known safe level of lead, the Lead and Copper Rule (LCR) established a lead concentration action limit of 15 parts per billion (ppb) and a maximum contaminant level goal (MCLG) of zero [20]. Following the Flint, Michigan water crisis, which was discovered in 2014, there was a renewed focus on lead exposure from drinking water [21]. For example, in 2019 Newark, New Jersey discovered elevated levels of lead in roughly 30 public schools and lead levels above 15 parts per billion in the city’s water [22]. Again in 2021, there was a renewed attention on lead in drinking water due to the Bipartisan Infrastructure Law and the allocation of $2.9 billion for the replacement of lead service lines in the United States [23]. 

While the risks of lead-contaminated water have long been understood, characterizing the distribution of lead contamination, particularly within vulnerable communities, has proven difficult. An environmental justice community is a community that is overburdened by hazardous waste, pollution, and increased vulnerabilities to hazards [24]. Environmental justice communities routinely express their distrust for government agencies, academic institutions, and organizations outside of their social and geographic boundaries [25,26]. This research leveraged a multi-year relationship with community members and local civic groups and utilized participatory-based research and community engagement methods to better respond to communal needs expressed by residents. This study provides evidence of lead-contaminated water in the neighborhood of the Greater Fifth Ward and the need for drinking water testing in low socioeconomic communities. It further demonstrates the importance of community-involvement when conducting environmental health research.

The neighborhood of the Greater Fifth Ward receives its water from the City of Houston, which serves approximately 2.2 million residents by operating three purification plants, 40 groundwater plants, and 16 supplemental groundwater plants [27]. The Texas Commission on Environmental Quality (TCEQ) classifies the City of Houston’s drinking water system as a “Superior Water Supply System,” which denotes the highest quality rating for water utility systems [27]. This indicates that the potential source of contamination is likely from pipes, faucets, and fixtures abutting or within the home. The 2020 Houston Water Quality Report indicates that of the homes monitored, customers’ tap water was roughly 90% below 4.01 ppb, and two of the samples were above 15 ppb [28]. Testing of the main water system detected an average of 0.2 ppb and a maximum of 1.4 ppb in samples [28]. Despite the high quality of water and purification methods used by the City of Houston, resident perceptions of water quality differ from actual results.

Previous studies that have investigated residents’ perception of tap water quality found that, although bottled water is less regulated, most households perceive bottled water to be of higher quality [29,30]. Perceptions of unsafe drinking water can negatively impact households due to unnecessary expenditures on bottled water; conservative estimates indicate that households spend thousands of dollars per year on bottled water [31], and the substitution of water for sugar-sweetened beverages [32]. Other studies that have investigated the relationship between race and perception of drinking water safety found that of 1174 respondents, white participants (65%) were more likely to believe their drinking water is safe compared to black respondents (42%) [33]. Past studies that assessed lead exposure from drinking water in homes in a similar neighborhood found lead in 30.8% of homes ranging from 0.6 to 2.4 (µg/L), whereas a mean lead concentration was discovered in 1.3 μg/L across drinking fountains in public parks (*N* = 56) and a maximum of 8.0 μg/L [34,35]. This study investigated drinking water quality, year the residence was built, and resident perceptions of drinking water quality since residents expressed concerns about the hazards of lead exposure and the need for testing.

## 2. Methods and Materials

### 2.1. Study Location and Population

**Figure 1:**
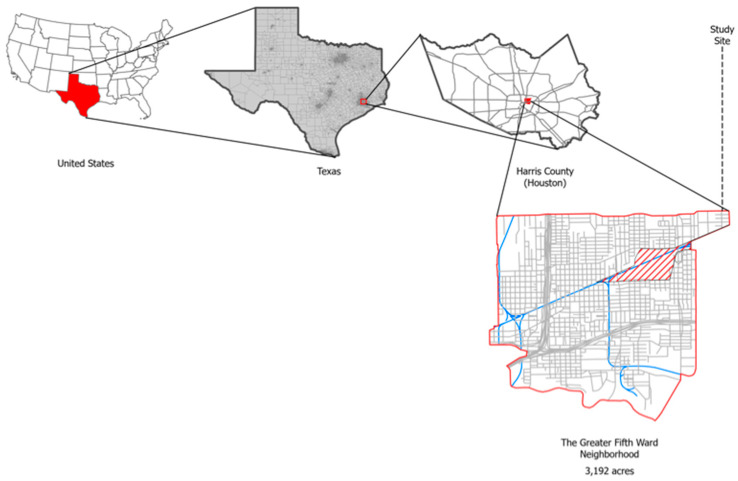
Study area in the neighborhood of the Greater Fifth Ward in Houston, TX, USA.

The Greater Fifth Ward is a geographically defined Super Neighborhood located east of downtown Houston, Texas (Figure 1). The city of Houston was divided into 88 super neighborhoods to encourage communities to work together to identify and set priorities to advocate for issues that matter to them most [36]. The boundaries of the Greater Fifth Ward are delineated by the Buffalo Bayou in the south, Lockwood drive on the east, Liberty road on the north, and Jensen drive in the west. This community experiences chronic pollution, such as air, soil, and water pollution, poor housing, and an inadequately built environment [37]. The neighborhood is disproportionately exposed to several sources of pollution, such as the Union Pacific Englewood railyard that transects the neighborhood, several Superfund site within close proximity, concrete crushing, and metallurgic recycling. The Greater Fifth Ward is a majority-minority neighborhood, given minorities make up a majority of the population. The population of the Greater Fifth Ward is 22,211 and is a predominately 63% African American and 35% non-white Hispanic community [38]. The average household income is $42,000, and roughly 4.7% (5386) live below the poverty line [39]. This neighborhood gained national attention in 2020 and 2021 when an assessment of the occurrence of cancer conducted by the Texas Department of State Health Services determined that the rates for acute myeloid leukemia (observed 56, standardized incidence ratio (SIR): 1.39, 95% CI: 1.05, 1.81), lung and bronchus (observed 478, SIR: 1.36, 95% CI: 0.83, 1.24, 1.49), esophagus (40 observed, SIR: 1.63, 95% CI: 1.16, 2.22) larynx (observed 53, SIR: 1.90, 95% CI: 1.42, 2.48), liver cancers (observed 275, SIR: 1.52, 95% CI: 1.35, 1.71), and childhood acute lymphoblastic leukemia were significantly greater than could be expected [40]. In 2019, the Houston Health Department reported that at least 6% of children living near Northside and the Greater Fifth Ward have blood lead levels above the Center of Disease Control and Prevention reference value of 5 micrograms per deciliter (µg/dL) [41].

### 2.2. Participatory-Based Training

Extensive training was held before deploying the community block captains and individuals from Texas A&M University. The block captains were recruited by a civic community group, the Coalition for Community Organizations (C.O.C.O.), whose mission is to educate, inform, and empower people. The block captains live in different geographic areas of the Greater Fifth Ward and are committed to improving issues that negatively impact their community. The block captains received specialized training via Zoom, pre-recorded videos demonstrating environmental sampling techniques, and participated in several in-person training days. The community block captains practiced sampling their own homes approximately nine months before sampling occurred in the wider community. This allowed the block captains to communicate to their neighbors and other residents about the experience. The block captains greatly contributed to the success of the study and were essential for executing community outreach efforts. Before the project began, the community block captains distributed door hangers, spoke to residents about the hazards of lead exposure, and informed them about the efforts our research team planned to undertake. The block captains reached the community by frequenting grocery stores, walking neighborhoods, and by attending community-sponsored events. The participants from Texas A&M University received training on cultural competency, proper interview techniques, how to complete contact forms, chains of custody, and environmental sampling. The instruction was delivered in person, which allowed for the demonstration and practice of the sampling methods. Before the teams deployed, a just-in-time training was given at the beginning of each trip. The individuals from Texas A&M University were instructed to rely on the local knowledge and expertise of the community block captains.

### 2.3. Household Survey

Research teams comprised field-trained community block captains from the neighborhood and individuals from Texas A&M University. Each group had at least one individual who could speak Spanish and was deployed from July to November 2021 for data collection. A complete canvasing method was attempted in the neighborhood of the Greater Fifth Ward. All residents aged 18 years and older were asked to participate; if the household was inaccessible, deemed unsafe, or had a no trespassing signage, field teams did not approach the home. A thirty-eight-question survey instrument was used to obtain demographic information, health history, health behaviors, and environmental risk perceptions. The survey instrument asked residents about the year their residence was built and if it had undergone any major renovations. Further, residents were asked if poor water quality and lead in the environment were issues in their community and were asked to rate those issues as excellent, very good, good, fair, and poor. The residents were also asked, “How concerned are you about any environmental pollution or contaminants in your community?”. The survey response options for this question included extremely, quite a bit, moderately, a little bit, and not at all. These questions were asked to get a better understanding of the residents’ perception of their water quality compared to reality. The survey included a question to determine the year their residence was built. The survey was developed in collaboration with the community partners. Participants received a $10 gift card for participating in the survey and environmental sampling. The survey instrument was approved by the Texas A&M University Institutional Review Board (IRB2021-0357D).

### 2.4. Water Sample Collection

Water samples were collected in the Greater Fifth Ward from residents’ kitchen taps by the residents themselves. The residents were asked to don a new pair of powder-free nitrile gloves and fill a 250 mL high-density polyethylene (HDPE) container with water from the hot water tap. Residents were instructed not to wait for the water to become hot before filling the container. The residents were asked to secure the lid of the container before returning it to the collection team. The collection team wore a new pair of nitrile gloves for each sample collected to ensure cross-contamination did not occur during the sample handling. Samples were placed into a designated sample cooler and remained in the cooler until they were transferred to the laboratory. The samples were stored in a freezer before analysis within the required timeframe for heavy metal samples.

### 2.5. Water Sample Analysis

The water samples were transported to the Trace Element Research Laboratory (TERL) at Texas A&M University Veterinary Medicine & Biomedical Sciences in College Station, Texas, where they were preserved to a pH of <2 with OmniTrace nitric acid (EMD Millipore Corp., Burlington, MA, USA). The water samples were analyzed by the United States Environmental Protection Agency method 200.8 (the Determination of Trace Elements in Waters and Wastes by Inductively Coupled Plasma-Mass Spectrometry) using a Perkin Elmer NexION 2000C instrument (PerkinElmer, Munich, Germany) quipped with a microconcentric nebulizer and a cyclonic spray chamber. Quality control was assured by blanks, laboratory control samples, standard reference materials (NIST 1640a, Trace Elements in Natural Waters), duplicate samples, and spiked samples. The limit of detection was 0.01 ug/L.

### 2.6. Data Analysis

Descriptive statistics, including the mean, standard deviation (SD), median, and interquartile range (IQR), were calculated for demographics, including sex, race/ethnicity, language, environmental and drinking water perception, the year the residence was built, and major renovation. The Generalized Linear Model (GLM) was applied to assess the statistical difference in the lead levels of tap water across the categories of each variable. Because the data on lead levels in drinking water was right-skewed, they were log-transformed before analysis. Tukey’s method was used for multiple comparisons. In additional analysis focusing on homes of 99 residents who reported both the year their home was built and major renovation afterward, the lead levels were compared across three categories: residences built before 1978 without major renovation afterward, residences built before 1978 with major renovation after 1978, and residences built after 1978. A box plot was used to present the lead concentrations in each category. Statistics were calculated using STATA 17 (StataCorp LLC, College Station, TX, USA). Statistical significance was declared at *p*-value < 0.05.

## 3. Results and Discussion

Among 175 residents who completed surveys, the homes of 172 residents (98.3%) had detectable levels of lead in their drinking water. Among those 172 residents, 55.7% were Black/African American (*N* = 98), 35.1% (*N* = 62) were Hispanic, and 22.8% (*N* = 13) were non-Hispanic White and others. 58.2% (*N* = 99) of the participants thought water quality was an issue, 49.4% (*N* = 87) thought that lead in the environment was a potential health issue for their families, and 70 residents (42.9%) rated it as poor. Among 101 residents who reported the year their residential homes were built, 43.6% of residents’ homes were built post-1978 (*N* = 44); however, 55.3% of the residential homes were built pre-1978 (56.4%, *N* = 57). Among the residential homes built pre-1978, 42.1% had major renovations after 1978 (doors/windows, pipe replacements, and/or any painting) (Table 1).

The mean of detectable lead levels in the 172 homes was 0.57 µg/L (95% CI 0.28–0.86). The results of the drinking water quality indicated concentrations of greater than 1 µg/L of lead in 10.9% (*N* = 19) of the samples. One of the homes had 22 µg/L concentrations of lead in the water and exceeded the United States Environmental Protection Agency action limit of 15 µg/L. This home was in the northern quadrant of the Greater Fifth Ward off the main thoroughfare. The results from the univariate analysis of water lead levels by characteristics, perceptions, and residential status are presented in Table 1.

Based on Tukey’s pairwise comparisons after a GLM, the average lead levels at homes without major renovations after 1978 were significantly higher than those at homes with major renovations among homes built pre-1978 (Table 2). However, there were no other significant differences in lead levels by demographics, perceptions, and year the residents were built.

**Figure 2:**
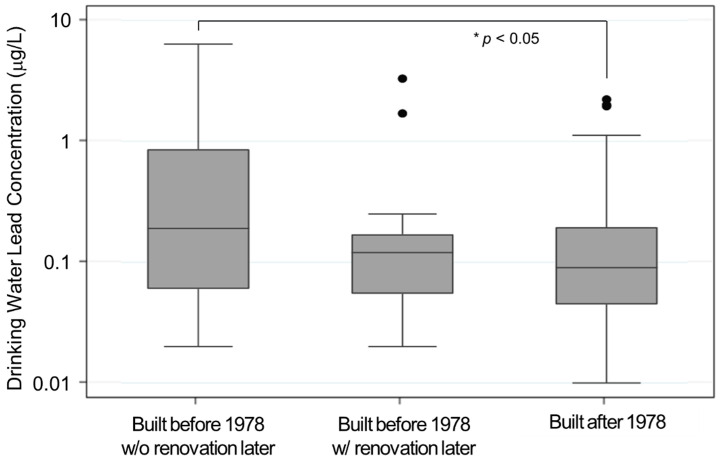
Log scale of lead concentrations (µg/L) in drinking water at 99 homes by year of residence built and major renovation after 1978. * Statistical significance at *p*-value < 0.05.

Although significant differences in lead levels were not found between homes built before and after 1978, 43.6% of homes built pre-1978 had major renovations afterward (Table 1 and Table 2). Therefore, an additional analysis was conducted focusing on homes of 99 residents who reported both the year their home was built and major renovation after 1978. The homes built prior to 1978 without major renovations after 1978 had significantly higher levels of drinking water lead compared to homes built after 1978 (Figure 2). The averages of lead concentrations were 0.75 µg/L (SD 1.28, IQR 0.06–0.86) at homes built before 1978 without major renovation after 1978, (*N* = 31), 0.31 µg/L (SD 0.72, IQR 0.06–0.17) at homes built before 1978 with major renovation after 1978 (*N* = 24), and 0.29 µg/L (SD 0.53, IQR 0.05–0.20) at homes built after 1978 (*N* = 44).

The results of the water analysis for each home were released to the residents accompanied by informational material about the associated adverse health effects. The homes with any detectable concentrations of lead in their drinking water were given a carbon filter to attach to their faucet.

## 4. Discussion

Engaging residents and community partners from the onset of the study was imperative to the project’s success. Prior to launching the project, we met with the founder of the Coalition of Community Organizations (C.O.C.O.) to sign an agreed-upon covenant with the community. The covenant outlined the roles and responsibilities of equally the Texas A&M University researchers and the community-based organization. The principal investigators agreed to maintain ethical research practices, articulate, and share research objectives and outcomes, share resources, and share aggregate data, whereas the founder of the Coalition of Community Organizations (C.O.C.O.) was requested to assist with connections to individuals and networks of people in the community, assisted with coordinating tasks/activities, and maintained active communication. Data were provided in the aggregate to partnered organizations along with a supply of drinking water filters that they could hand out to anyone in the community who requested one.

Preparing the community block captains to speak about the harms and health hazards of lead exposure, environmental contaminants, and environmental sampling primed the larger community for the sampling efforts. They had, in addition to this project, been active with other academic institutions and environmental advocacy groups, such as the Texas Southern University (TSU) and the Environmental Defense Fund (EDF). Further, the block captains engaged in outreach activities at community events, such as grocery stores, neighborhoods, and movie drive-ins, to bring awareness and concern about the hazards of lead, and to inform the residents that a sampling campaign would be underway. The trained community block captains opened doors, gained trust with residents, and greatly increased resident participation compared to past efforts. The benefits of community partnerships are well documented, and the merits of participatory-based research are encouraged by research institutions [42]. The biggest apprehension expressed by our community partners’ was the fear that our partnership was not long-term; therefore, it was essential to keep open lines of communication and pursue funding opportunities for future research. The trusting relationship developed during this project has allowed for expanded opportunities for research and intervention beyond the scope of this study. After completion of the study, the results will be communicated to community partners and residents of the Greater Fifth Ward in appropriate and understandable terms. The block captains expressed interest in jointly reporting the results of the study during a town hall and requested that it is offered both in-person and online. Disseminating the results to both the study participants and the wider community was intended to uphold ethical, cultural, and scholarly principles outlined at the beginning of our partnerships. This research was responsive to community desires and set the stage for future initiatives and efforts to identify potential causal impacts on health within this community.

This project has several limitations. A key limitation is that, although the research teams deployed in the morning, it is not known if the drinking water samples collected from the residents’ kitchens were the first draw of the day. The residents were not contacted to participate in the study before the research teams deployed. Therefore, households may have been exposed to higher levels of lead at an earlier time of day. Although our research methods did not provide an opportunity to obtain the first draw of the day, there were still lead concentrations found in the water. Future research should consider sampling these homes again to determine exact concentrations. The survey questions were interviewer-administered, which may influence how a participant chooses to respond compared to a self-administered survey [43]. Other characteristics of interviewers, such as race, ethnicity, and gender, are also known to have interviewer effects on responses [44]. While the field teams did not approach homes that were considered unsafe, they may have missed residents that had differing views of environmental risks and contaminants. This issue may have been avoided by providing surveys through other means. Additional limitations of this study are that there may be other potential sources for lead that were not investigated and that blood samples were not collected from the households.

### Public Health Implications

This study demonstrates the value and need for community engagement and participatory research to address environmental health concerns. While all the samples, except one, were below the USEPA action level for lead concentrations, the onus to mitigate residential water infrastructure rests on the resident. The results further illustrate the importance of lead abatement programs and policies since homes built prior to 1978 without major renovations had significantly higher levels of lead in their drinking water compared to homes built after 1978. The Bipartisan Infrastructure Law may help fast-track the replacement of lead pipes, lead faucets, and fixtures in historically disadvantaged environmental justice communities. The use of carbon filters and water pitchers is highly recommended for residents with detectable levels of lead. Filtering the water will provide the residents with safe potable water and reduce potential exposures to hazardous contaminants. Further, knowing the environmental perceptions of lead risk has been shown to greatly change outcomes of risk management and willingness to follow mitigation strategies [45]. Results from the survey can help in crafting future intervention strategies by research, city officials, or community groups in attempting to reduce the risk of environmental pollutants in this vulnerable community.

## 5. Conclusions

Previous efforts within environmental justice communities in general [46], and the Greater Fifth Ward in particular [47], have found it difficult to collect environmental data within vulnerable minority communities. The approach we have taken here included collaborating across multiple organizations and providing residents with the tools and means to be a part of the research from conception to community outreach efforts and result dissemination. Additional efforts put towards training six teams of block captains recruited from the community opened communication between residents and the research team and played a large role in collecting data that individuals would otherwise not wish to provide.

## Figures and Tables

**Table 1:** Descriptive analysis of participants’ characteristics and water lead levels (*N* = 172).

Characteristics	*N* (%)	Lead Level (μg/L)		
		Mean (SD)	Median	IQR
**Gender**				
Male	67 (39.2)	0.44 (1.15)	0.10	0.05−0.23
Female	104 (60.8)	0.66 (2.31)	0.14	0.06−0.37
**Race and Ethnicity**				
Non-Hispanic Black	95 (56.5)	0.69 (2.47)	0.13	0.05−0.28
Hispanic	60 (35.7)	0.40 (0.97)	0.11	0.05−0.20
All others	13 (7.7)	0.53 (0.73)	0.17	0.06−0.80
**Language Preference**				
English	143 (83.1)	0.62 (2.11)	0.12	0.05–0.28
Spanish	29 (16.9)	0.30 (0.43)	0.12	0.04–0.40
**Perceived Water Quality**				
Yes	99 (58.2)	0.44 (1.06)	0.13	0.06−0.27
No	71 (41.8)	0.75 (2.74)	0.11	0.05−0.31
**Perceived Drinking Water Quality**				
Good or above	55 (33.7)	0.75 (2.99)	0.13	0.05−0.31
Fair	38 (23.3)	0.53 (1.16)	0.09	0.04−0.22
Poor	70 (42.9)	0.49 (1.22)	0.13	0.06−0.34
**Perceived Lead in the Environment**				
Yes	85 (49.4)	0.48 (1.16)	0.13	0.05−0.26
No	85 (49.4)	0.67 (2.49)	0.12	0.05−0.31
**Year Residence Built**				
Pre 1978	57 (56.4)	0.54 (1.07)	0.15	0.06−0.43
Post 1978	44 (43.6)	0.29 (0.53)	0.09	0.05−0.20
Major Renovations ** After 1978 (Pre-1978 Residences)				
Yes	24 (43.6)	0.31 (0.72)	0.12	0.06−0.17
No	31 (56.4)	0.75 (1.28)	0.15	0.06−0.86

**: Major renovations include doors/windows, pipe replacements, and/or any painting.

**Table 2:** Results from Tukey’s pairwise comparisons assessing statistical differences in water lead levels * by characteristics.

Characteristics	β Coefficient (95% CI)	*p*-Value
**Gender**		
Male vs. Female	−0.22 (−0.65, 0.21)	0.32
**Race and Ethnicity**		
Non-Hispanic Black vs. Hispanic	0.24 (−0.31, 0.78)	0.56
Non-Hispanic Black vs. All Others	−0.23 (−1.20, 0.75)	0.85
Hispanic vs. All Others	−0.46 (−1.47, 0.55)	0.52
**Language Preference**		
English vs. Spanish	0.20 (−0.36, 0.76)	0.49
**Perceived Water Quality**		
Yes vs. No	−0.02 (−0.45, 0.41)	0.93
**Perceived Drinking Water Quality**		
Good or above vs. Fair	0.16 (−0.54, 0.87)	0.85
Good or above vs. Poor	0.02 (−0.58, 0.62)	0.99
Fair vs. Poor	−0.14 (−0.81, 0.53)	0.87
**Perceived Lead in the Environment**		
Yes vs. No	0.02 (−0.41, 0.44)	0.94
**Year Residence Built**		
Post 1978 vs. Pre 1978	−0.53 (−1.08, 0.02)	0.05
Major Renovations ** After 1978 (Pre-1978 Residences)		
Yes vs. No	−0.73 (−1.48, −0.01)	0.04 ***

*: Lead levels were log-transformed before the analysis because they were right-skewed; **: Major renovations include doors/windows, pipe replacements, and/or any painting; ***: Statistically significant difference at *p*-value < 0.05.

## Data Availability

Data is available upon request.
